# Academic jealousy, attitudes, and motivation in relation to foreign language learning effort: a structural equation modeling approach

**DOI:** 10.3389/fpsyg.2026.1739546

**Published:** 2026-03-19

**Authors:** Burak Ayçiçek, Şenol Orakci

**Affiliations:** 1 Department of Educational Sciences, Faculty of Education, Tokat Gaziosmanpaşa University, Tokat, Türkiye; 2 Department of Educational Sciences, Faculty of Education, Aksaray University, Aksaray, Türkiye

**Keywords:** academic jealousy, attitude, foreign language learning effort, higher education, motivation

## Abstract

**Introduction:**

As the academic landscape of the 21st century has become increasingly competitive, emotion-related processes have attracted growing attention as potential influences on learners’ engagement in foreign language study. Accordingly, the current study aims to investigate the effect of academic jealousy of university students studying in the field of English on their foreign language learning efforts through attitude toward foreign language learning and motivation.

**Method:**

This study employed a cross-sectional survey model, a commonly used approach within quantitative research. The study sample comprised 360 university students enrolled in English language programs during the 2024–2025 academic year. Academic Jealousy Scale, Attitudes Toward Foreign Language Learning Scale, Motivation Scale Intended for Learning English, and Foreign Language Learning Effort Scale were used to collect data. The theoretical model of foreign language learning effort and the effects of variables were analyzed through structural equation modeling.

**Results:**

The findings indicated that academic jealousy levels of participants have a positive and significant effect on their attitudes toward foreign language learning and intrinsic motivations toward learning English. However, academic jealousy has a negative and significant effect on the levels of extrinsic motivation toward learning English. Furthermore, attitudes toward learning a foreign language, intrinsic and extrinsic motivation levels toward learning English were found to have a positive and significant effect on foreign language learning effort.

**Conclusion:**

Overall, the study suggests that academic jealousy is a meaningful emotional factor in English learning that relates to students’ attitudes, motivation, and sustained learning effort. These findings highlight the value of supportive, learning-focused instructional practices that reduce negative social comparison and foster more persistent engagement.

## 1 Introduction

Humans are inherently inclined to evaluate themselves through comparison with others, a process that profoundly shapes their emotions, behaviors, and motivational patterns. Social Comparison Theory posits that, in the absence of objective benchmarks, individuals assess their abilities and accomplishments by referencing those around them (Festinger, 1954). Such comparisons can yield diverse emotional and behavioral consequences—ranging from discouragement and diminished self-worth to increased motivation and self-improvement (Gupta et al., 2021). These evaluations frequently manifest as upward comparisons, in which individuals measure themselves against those perceived as more capable or successful (Buunk, 2024). However, persistent engagement in such comparisons and the emotional reactions they elicit may strain interpersonal relationships, impede communication, undermine teamwork, and ultimately erode both performance and wellbeing (Reyna, 2021).

In academic settings, these comparisons often manifest as academic jealousy (Anderson, 2002). Academic jealousy has been linked to increasing competition in institutions, driven by population growth and highly competitive work environments (Bayar and Koca, 2021). Jealousy arises when individuals perceive a threat to something they already possess such as a valued relationship or status, prompting efforts to protect or maintain it (Parrott, 1991). One critical subdimension of academic jealousy is envy, defined as emotional discomfort and hostility that emerge when individuals compare themselves unfavorably with others whom they perceive as more successful or advantaged (van de Ven, 2017). Envy is closely tied to upward social comparison and tends to arise when individuals feel they lack others’ traits or accomplishments (Carraturo et al., 2023; Smith and Kim, 2007; van de Ven, 2017). Prior work suggests that envy can sometimes enhance motivation and self-control, but it can also intensify negative outcomes such as stress, reduced focus, and lower productivity (Clanton, 2018; Taj et al., 2020). Envy may be experienced as benign envy, which motivates self-improvement, or malicious envy, which motivates efforts to undermine the target or devalue their success (van de Ven et al., 2009).

In educational environments, envy often arises through comparisons of grades, language proficiency, or accomplishments among peers (Utz and Muscanell, 2018). At moderate levels, envy can serve as a motivator, encouraging students to work toward self-improvement and achieve greater success. However, in most cases, envy’s detrimental effects—such as strained relationships and reduced collaboration—outweigh its benefits, particularly when driven by factors such as mental health conditions or bullying (Nurhayati et al., 2020; Reyna, 2021). Additionally, envy negatively affects self-regulation, making it more challenging for students to stay on track with their academic goals (Hill et al., 2011). In competitive academic environments, where students are expected to succeed while maintaining friendships and working with others, envy often develops as a result of these mixed expectations.

Benign envy has been shown to foster motivation and effort by encouraging students to work harder and improve performance (Hsiao et al., 2012; Nurhayati et al., 2020; Sitinjak, 2016). By contrast, malicious envy can disrupt motivation, self-regulation, and interpersonal relationships (Nurhayati et al., 2020; Taj et al., 2020; Toomey and Heo, 2022), and may lead to disengagement and reduced motivation (Toomey and Heo, 2022). Moreover, students experiencing malicious envy may focus on diminishing others’ achievements rather than self-improvement (Hill et al., 2011). These dynamics highlight the dual role of envy in shaping motivation and effort in educational contexts (von Kriegstein, 2017). Overall, envy, as a central emotional component of academic jealousy, can function both as a motivator and as a barrier to effective learning (Fisher and Ford, 1998; Hsiao et al., 2012).

Effort is a critical factor in academic success and involves the intentional allocation of mental energy to learning tasks (Fisher and Ford, 1998; von Kriegstein, 2017). When students are made aware of their peers’ progress, upward comparisons can motivate them to increase their effort, particularly in competitive environments (Hsiao et al., 2012). However, without sufficient motivation, students may struggle to maintain the focus and effort needed to achieve their academic goals (Bandhu et al., 2024; Tohidi and Jabbari, 2012).

In educational settings, students often engage in social comparisons, evaluating academic performance, physical appearance, and athletic abilities (Fleur et al., 2023). Such comparisons can motivate students (Chen and Chen, 2023) and encourage them to strive for similar accomplishments. They may also enhance cognitive engagement and learning outcomes (Wambsganss et al., 2022). Social comparisons shape individuals’ perceptions of their future selves, goals, and actions. Both positive and negative role models can serve as benchmarks for challenging objectives, such as academic success or career advancement, and may help sustain behavior change over time (Lockwood and Pinkus, 2008). However, excessive comparison can also elicit dissatisfaction, guilt, and regret, thereby impeding progress and wellbeing (Allan and Gilbert, 1995).

In the context of second language acquisition, social comparisons among learners can influence motivation and shape attitudes toward language learning (Hong et al., 2022). A range of emotions (e.g., fear, worry, excitement, and happiness) has been linked to learners’ engagement and attitudes (Tingyu et al., 2024; Wang et al., 2024), and peer comparison is a prominent trigger of these emotional experiences (Richards, 2022). Differences in learners’ language abilities within a group often prompt comparisons that may increase anxiety, envy, fear, and reluctance to participate (Zhang and Head, 2010). At the same time, peer comparison may motivate learners to match classmates’ proficiency and invest greater effort (Yan and Horwitz, 2008). Consistent with this view, learners who rely on social comparison may increase engagement to learn from more experienced peers (Neugebauer et al., 2016), and students who compare their grades with higher-performing peers may report greater motivation to exert effort in subsequent tasks (Vázquez et al., 2023). Thus, social comparison can stimulate effort and motivation, but it can also intensify unpleasant emotions that hinder achievement.

**FIGURE 1:**
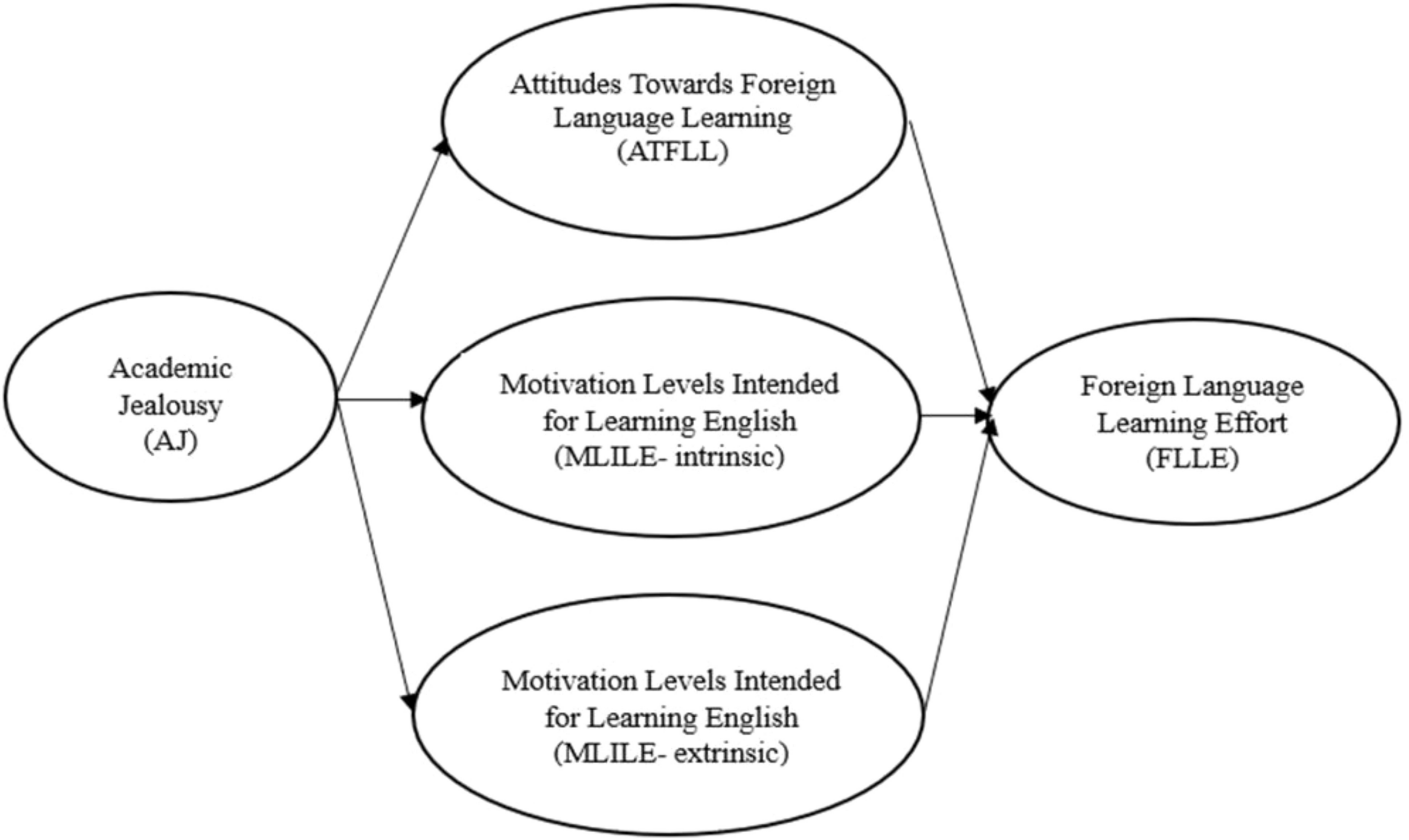
A model of foreign language learning efforts of students.

Peer effects further influence students’ attitudes, both directly and indirectly. Direct peer effects may include collaborative learning or tutoring, in which students actively support one another. Indirectly, peers’ attitudes, interests, and motivation can create an environment that inspires or discourages learners (Jin et al., 2022). Given the role of attitudes in shaping engagement and success, attitudes have been emphasized as an important component of second or foreign language pedagogy (Getie, 2020). Building on this insight, the present study examines how academic jealousy among university students enrolled in English-related programs influences foreign language learning effort through attitudes toward foreign language learning and motivation, using structural equation modeling (SEM). Although the broader term foreign language learning is used for conceptual framing, the target language examined in the present study is specified as English. The conceptual framework of this study is illustrated in Figure 1. The hypotheses guiding the study are presented below.

H_1_: AJ of students affects ATFLL positively.

H_2_: AJ of students affects MLILE- intrinsic positively.

H_3_: AJ of students affects MLILE- extrinsic positively.

H_4_: ATFLL of students affects FLLE positively.

H_5_: MLILE- intrinsic of students affects FLLE positively.

H_6_: MLILE- extrinsic of students affects FLLE positively.

## 2 Research design

This research employed a cross-sectional survey design, a commonly used quantitative approach for examining participants’ current attitudes, perceptions, or beliefs at a specific point in time (Creswell, 2014; Fraenkel and Wallen, 2009). This design was considered as appropriate as it allows for the description of the existing conditions of the sample within a defined timeframe, facilitates the generalization of findings to a broader population, accommodates variation across developmental stages such as grade, and supports data collection at a single time point.

### 2.1 Study group

The population of this study consists of university students studying in the field of English in eight different state universities in Türkiye. The sample comprised 360 students enrolled at various grade levels across the selected universities during the 2024–2025 academic year. The convenience sampling method was preferred in the selection of the participants. The necessary ethical approvals were secured for the study. The year and protocol number of the document is 2024/03-160. After the students were informed about the study, those who voluntarily participated in the study constituted the sample group. Demographic information regarding the participants is presented in Table 1.

**TABLE 1:** Demographic information of the sample group.

Variables	Category	*N* = 360	%
Gender	Female	263	73.1
	Male	97	26.9
Age	18–21	157	43.6
	22–25	182	50.6
	Over 25	21	5.8
Grade	1st year	28	7.8
	2nd year	82	22.8
	3rd year	85	23.6
	4th year	165	45.8
Department	English language and literature	215	59.7
	English language teaching	145	40.3
Other foreign language	German	204	57.7
	French	47	13.1
	Russian	17	4.72
	Spanish	15	4.2
	Arabic	9	2.5
	Japanese	8	2.2
	Dutch	4	1.11
	Italian	3	0.8
	Korean	2	0.6
	None	59	16.4

As presented in Table 1, the research sample consists of 360 university students, of whom 263 are female and 97 are male. There are 157 students between the ages of 18–21, 182 students between the ages of 22–25, and 21 students over the age of 25 in the sample. A total of 28 of the students were in the 1st year, 82 in the 2nd year, 85 in the 3rd year, and 165 in the 4th year. The study includes 215 students from the Department of English Language and Literature and 145 students from the Department of English Language Teaching. Apart from English, students know nine other foreign languages. The most commonly known foreign language, other than English, is German, spoken by 204 students. Six students have more than one foreign language other than English. In addition, 59 students do not have any other foreign language other than English.

### 2.2 Data collection tools

The demographic information, Academic Jealousy Scale, Attitudes Toward Foreign Language Learning Scale, Motivation Scale Intended for Learning English, and Foreign Language Learning Effort Scale were used to collect data. The reliability of the sub-dimensions of the scales was analyzed with Cronbach’s alpha coefficient and McDonald’s omega value, and the reliability of the whole scale was analyzed with stratified alpha and Revelle’s omega values. Cronbach’s alpha coefficient and McDonald’s omega are expected to be above 0.70 in order for the scale to be considered reliable in terms of internal consistency (Hair et al., 2005; Nunnally and Bernstein, 1994). For stratified alpha and Revelle’s omega, values above 0.80 indicate that the total scores obtained from the multidimensional scale are reliable (Nájera Catalán, 2019). The construct validity of the scales was tested by confirmatory factor analysis (CFA). The fit indices calculated with CFA were interpreted by considering the ranges recommended by Chan and Sun (2019), Kline (2016), and Tabachnick and Fidell (2013). Data were collected online via Google Forms.

Demographic Information Form: The form consists of five items including the student’s gender, age, grade, department, and foreign language other than English.

Academic Jealousy Scale: The scale was developed by Koçak (2019). It consists of 19 items, including three sub-dimensions as Envy (7 items), Self-denigration (6 items), Maturity (6 items). Scale items are in five-point Likert type as (1) never, (2) rarely, (3) sometimes, (4) usually, (5) always. The scale includes three reverse-coded items (items 2, 13, and 14). Scores on the scale range from a minimum of 19 to a maximum of 95. In the present study, the mean score was 71.18 (sd = 8.49), and the three-factor structure accounted for 50.80% of the total variance. The reliability analysis resulted in Cronbach’s alpha values of 0.83, 0.80, and 0.70 for the sub-dimensions, and McDonald’s omega values were 0.85, 0.81, and 0.73, respectively. Therefore, it is found that all sub-dimensions of the scale are reliable. The stratified alpha coefficient for the whole scale was 0.77 and Revelle’s omega coefficient was 0.86. Accordingly, it can be said that the total scores obtained from the scale are reliable. As a result of CFA with four modifications, fit indices were calculated as χ^2^[145, *N* = 360] = 384.831, *p* < 0.01; χ^2^/df = 2.65; CFI = 0.90; GFI = 0.90; SRMR = 0.078; RMSEA = 0.068. Therefore, the three-factor model demonstrated an acceptable fit to the data.

Attitudes Toward Foreign Language Learning Scale: The scale was developed by Adıyaman (2019). It consists of 16 items, including three sub-dimensions as Interest (9 items), Utility (4 items), Concern (3 items). The items in the scale are in a five-point Likert scale with (1) strongly disagree, (2) slightly agree, (3) somewhat agree, (4) mostly agree, (5) completely agree. Of these items, ten are positively formulated, while six (items 2, 5, 8, 9, 10, and 16) are negatively formulated. Scores on the scale range from a minimum of 16 to a maximum of 80. In the present study, the mean score was 69.66 (sd = 5.12), and the three-factor structure accounted for 45.71% of the total variance. The reliability analysis resulted in Cronbach’s alpha values of 0.78, 0.61, and 0.71 for the sub-dimensions, and McDonald’s omega values were 0.80, 0.65, and 0.71, respectively. Therefore, it is found that the reliability in the Utility sub-dimension of the scale is weak, while the other sub-dimensions are reliable. The stratified alpha coefficient for the whole scale was 0.72 and Revelle’s omega coefficient was 0.83. Accordingly, it can be said that the total scores obtained from the scale are reliable. As a result of CFA with two modifications, fit indices were calculated as χ^2^[99, *N* = 360] = 299.564, *p* < 0.01; χ^2^/df = 3.03; CFI = 0.85; GFI = 0.90; SRMR = 0.066; RMSEA = 0.075. CFI between 0.80 and 0.89 indicates acceptable fit (Bentler, 1990). Accordingly, the three-factor model demonstrated an acceptable fit to the data.

Motivation Scale Intended for Learning English: The scale was developed by Karayazgan and Saracaloğlu (2021). It consists of 32 items, including two sub-dimensions as Intrinsic Motivation (19 items) and Extrinsic Motivation (13 items). The items in the scale are in five-point Likert type as (1) strongly disagree, (2) disagree, (3) partially agree, (4) agree, (5) strongly agree. Of these items, twenty-nine are positively formulated, while three (items 10, 12, 13) are negatively formulated. Scores on the scale range from a minimum of 32 to a maximum of 160. In the present study, the mean score was 125.33 (sd = 15.40), and the two-factor structure accounted for 40.78% of the total variance. The reliability analysis resulted in Cronbach’s alpha values of 0.88 and 0.89 for the sub-dimensions, and McDonald’s omega values were 0.90 and 0.89, respectively. Thus, it is found that both sub-dimensions of the scale have high reliability. The stratified alpha coefficient for the whole scale was 0.88 and Revelle’s omega coefficient was found to be 0.92. Accordingly, it can be said that the total scores obtained from the scale are reliable. As a result of CFA with six modifications, fit indices were calculated as χ^2^[457, *N* = 360] = 1,486.916, *p* < 0.01; χ^2^/df = 3.25; CFI = 0.81; GFI = 0.86; SRMR = 0.077; RMSEA = 0.079. GFI between 0.85 and 0.89 is considered as acceptable fit (Jöreskog and Sörbom, 1993). In addition, CFI between 0.80 and 0.89 indicates acceptable fit (Bentler, 1990). Accordingly, the two-factor model demonstrated an acceptable fit to the data.

Foreign Language Learning Effort Scale: The scale was developed by Karabıyık and Mirici (2018). It consists of 17 items, including four sub-dimensions as Non-complaince (3 items), Procedural (3 items), Substantive (8 items), Focal (3 items). The items in the scale are in five-point Likert type as (1) never, (2) rarely, (3) sometimes, (4) often, (5) always. Of these items, fourteen are positively formulated, while three (items 2, 8, 14) are negatively formulated. Scores on the scale range from a minimum of 17 to a maximum of 85. In the present study, the mean score was 66.44 (sd = 9.24), and the four-factor structure accounted for 56.61% of the total variance. The reliability analysis resulted in Cronbach’s alpha values of 0.65, 0.72, 0.79, and 0.70 for the sub-dimensions, and McDonald’s omega values were 0.69, 0.74, 0.80, and 0.72, respectively. Therefore, it is found that the reliability in the Non-complaince sub-dimension of the scale is weak, while the other sub-dimensions are reliable. The stratified alpha coefficient for the whole scale was found to be 0.87 and Revelle’s omega coefficient was found to be 0.90. Accordingly, it can be said that the total scores obtained from the scale are reliable. As a result of CFA, fit indices were calculated as χ^2^[113, *N* = 360] = 438.624, *p* < 0.01; χ^2^/df = 3.88; CFI = 0.83; GFI = 0.87; SRMR = 0.072; RMSEA = 0.078. Accordingly, the four-factor model demonstrated an acceptable fit to the data.

### 2.3 Data analysis

In the study, missing data and outliers were first analyzed, and then SEM assumptions such as sample size, normality, linearity, multicollinearity and singularity were tested (Kline, 2016). The dataset, comprising responses from 372 university students collected via Google Forms, contained no missing values. Outliers were identified through standardized z-scores, and 12 outliers with values falling outside the range of [−3, 3] were excluded from the dataset (Tabachnick and Fidell, 2013), resulting in a final sample of 360 students. Additionally, a power analysis was conducted using G*Power (v3.1.9.7) to determine the adequacy of the sample size. Based on the criteria suggested by Faul et al. (2007), the analysis indicated that a minimum sample size of 291 was required to achieve a power of 0.95, an alpha of 0.05, and a small effect size (f^2^ = 0.06$). In addition, Kaiser–Mayer Olkin (KMO) test was performed for the adequacy of the sample size and the results were in the range of 0.813 to 0.884, which was interpreted that the sample of 360 students was sufficient (Kaiser, 1970). The fact that the calculated skewness and kurtosis coefficients are in the range of −1.5 and 1.5 indicates that the univariate normality of the data is provided, and the Bartlett’s Sphericity test results are significant (*p* < 0.001) indicates that multivariate normality is provided (Tabachnick and Fidell, 2013). The residual graphs were analyzed to check the linearity assumption and the random distribution of the residuals against the predicted values was interpreted as the model met the linearity assumption. Tolerance and variable inflation factor (VIF) values regarding the multicollinearity assumption were analyzed. It was observed that tolerance values ranged between 0.68 and 0.83 and VIF values ranged between 1.200 and 1.478. Tolerance values above 0.10 and VIF values less than 10 indicate that there is no multicollinearity problem in the data (Kline, 2016).

The theoretical model of foreign language learning effort and the effects of variables were analyzed through SEM. Although the internal consistency coefficients were low in some sub-dimensions of the scales, the reliability of the total scores was high and the theoretical model was constructed based on the total scores. Maximum likelihood-structural equation modeling (ML-SEM) was used in the current study in which continuous variables were used and normality assumptions were met (Hair et al., 2012). During the analysis, the validity of the measurement model—formulated in accordance with the theoretical framework—was initially evaluated, after which the structural model was tested to examine the research hypotheses. In the evaluation of model fit, χ^2^ and *p*-value, χ^2^/df, CFI, GFI, SRMR, and RMSEA values were used. IBM SPSS Statistics 22, Jamovi 2.3.26, and IBM SPSS AMOS 22 were used in the analyses.

## 3 Results

In the study, a SEM was constructed regarding the foreign language learning efforts of university students. Firstly, when the fit indices (χ^2^ [449, *N* = 360] = 1,218.120; *p* < 0.01; χ^2^/df = 2.713; CFI = 0.86; GFI = 0.88; SRMR = 0.072; RMSEA = 0.069) calculated with three modifications are examined, it is found that the measurement model demonstrated an acceptable level of fit to the data and was considered as valid. Subsequently, the structural model was employed to test the research hypotheses. The results of this analysis are given in Table 2.

**TABLE 2:** Results of the SEM.

Paths	β	CR	*P*	Hypotheses	Hypothesis results
AJ → ATFLL	0.35	3.90	< 0.001	H_1_	Supported
AJ → MLILE-intrinsic	0.22	3.11	< 0.01	H_2_	Supported
AJ → MLILE-extrinsic	−0.35	−3.25	< 0.001	H_3_	Not supported
ATFLL → FLLE	0.50	4.45	< 0.001	H_4_	Supported
MLILE-intrinsic → FLLE	0.21	2.61	< 0.01	H_5_	Supported
MLILE-extrinsic → FLLE	0.15	2.41	< 0.05	H_6_	Supported

β, standardized path coefficient; CR, critical ratio.

According to Table 2, the effect of academic jealousy (AJ) on attitude toward foreign language learning (ATFLL) (β = 0.35; *p* < 0.001), on intrinsic motivation level toward learning English (MLILE-intrinsic) (β = 0.22; *p* < 0.01) and on extrinsic motivation level toward learning English (MLILE-extrinsic) (β = −0.35; *p* < 0.001) is significant. Similarly, the effect of attitude toward foreign language learning (ATFLL) on foreign language learning effort (FLLE) (β = 0.50; *p* < 0.001), the effect of intrinsic motivation level toward learning English (MLILE-intrinsic) on foreign language learning effort (FLLE) (β = 0.21; *p* < 0.01) and the effect of extrinsic motivation level toward learning English (MLILE-extrinsic) on foreign language learning effort (FLLE) (β = 0.15; *p* < 0.05) are also significant. When the Critical Ratio (CR) value, which is used to evaluate the statistical significance of the parameters in a model, exceeds ∓1.96, the relevant parameter is considered to be statistically significant at *p* < 0.05. In this case, the null hypothesis is rejected (Khine, 2013). Since the CR values exceed 1.96, all null hypotheses are rejected.

**FIGURE 2:**
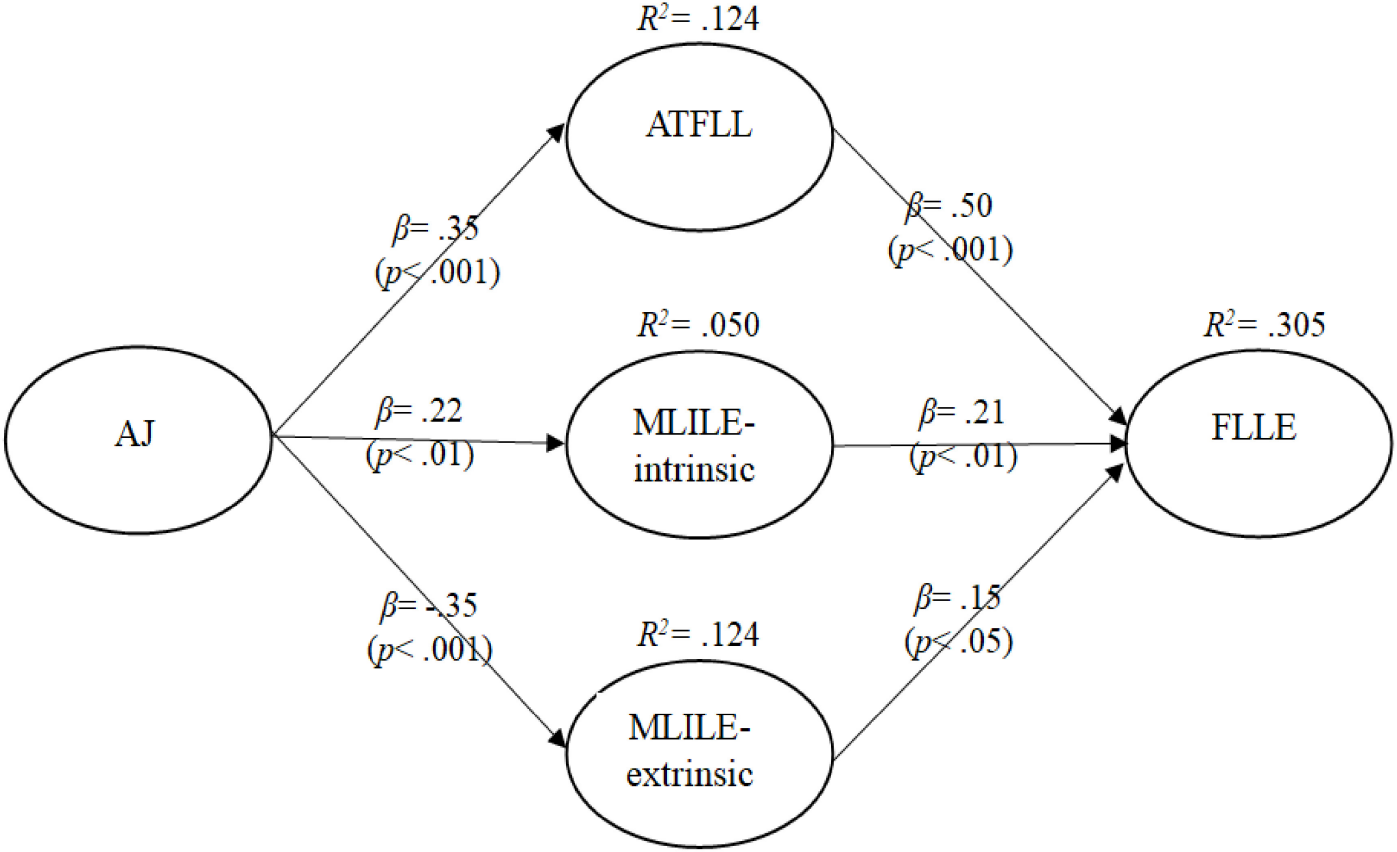
The structural model related to foreign language learning effort.

The path coefficients between the latent variables and the structural model of foreign language learning effort are presented in Figure 2.

When Figure 2 is analyzed, it is seen that, in line with the research hypotheses, academic jealousy levels of university students have a positive and significant effect on their attitudes toward foreign language learning and intrinsic motivations toward learning English. Therefore, H1 and H2 hypotheses were supported. However, academic jealousy has a negative and significant effect on the levels of extrinsic motivation toward learning English. Therefore, hypothesis H3 was not supported. Contrary to expectations, an increase in students’ academic jealousy levels decreases their extrinsic motivation to learn English. It is found that academic jealousy is more effective on attitudes toward foreign language learning and extrinsic motivation toward learning English. In addition, attitudes toward learning a foreign language, intrinsic and extrinsic motivation levels toward learning English were found to have a positive and significant effect on foreign language learning effort. Therefore, hypotheses H4, H5 and H6 were supported. Compared to the others, it is seen that attitude toward foreign language learning is more effective on foreign language learning effort.

In the constructed model, academic jealousy explains approximately 12% of the variance in attitudes toward foreign language learning, 5% of the variance in intrinsic motivation to learn English, and 12% of the variance in extrinsic motivation to learn English. Moreover, the constructed structural model explains approximately 31% of the variance in foreign language learning effort. Therefore, it can be concluded that the structural model created based on literature is an explanatory and conceptually integrated model of foreign language learning effort.

## 4 Discussion and conclusion

The findings show that academic jealousy significantly affects attitudes toward foreign language learning, as well as intrinsic and extrinsic motivation for learning English. Academic jealousy has a positive and statistically significant effect on attitudes toward foreign language learning, suggesting that students who experience such emotions may develop more favorable perceptions of language learning. This may reflect the competitive nature of language acquisition, in which learners assess their performance against peers and strive to become more proficient. In this context, jealousy may function as a motivating force rather than solely a negative emotion.

As contemporary society increasingly emphasizes competition and material success, young people may feel pressure to compete. When they fall short of others’ standards, jealousy may increase and self-esteem may decline (Sheldon et al., 2001). At the same time, perceiving peers as more skilled may prompt students to improve, which can result in more positive attitudes toward learning. This interpretation aligns with social comparison theory and evidence from competitive learning environments, showing that competition with similar or slightly more capable peers can enhance motivation and performance (Collins, 1996; Munkes and Diehl, 2003; Tor and Garcia, 2023; Wang et al., 2023). In language learning contexts, students may therefore strive to develop proficiency when they perceive classmates as more skilled.

The significant positive effect of academic jealousy on intrinsic motivation suggests that certain students may use their jealousy as a driving force to achieve fluency in the language. Intrinsically motivated learners engage in learning activities because they find them interesting and enjoyable, rather than for external rewards (Di Domenico and Ryan, 2017). When jealousy arises from observing more proficient peers, it may ignite a personal challenge to improve, thereby enhancing self-directed learning behaviors. This finding aligns with the self-determination theory, which posits that intrinsic motivation is strengthened when learners experience a sense of competence and autonomy (Wang et al., 2019).

Interestingly, the negative effect of academic jealousy on extrinsic motivation suggests that students experiencing jealousy may become less driven by external rewards, such as grades, approval, or social recognition. It can be argued that jealousy, as an upward social-comparison emotion, can elicit feelings of threat or discouragement and thereby reduce the perceived attainability or value of externally regulated goals. Accordingly, when jealousy becomes salient in academic comparison contexts, learners may become less responsive to reward-contingent motives. Consequently, this situation may reflect disengagement from reward-based learning behaviors, particularly when comparisons become overwhelming and elicit frustration or self-doubt. In such cases, peers’ success may be perceived as unattainable, which can further reduce motivation to meet external standards of achievement.

According to the findings, students’ effort in foreign language learning is positively and significantly influenced by their attitudes toward foreign language learning, as well as by both intrinsic and extrinsic motivation to learn English. These findings highlight the crucial role of motivational and attitudinal factors in shaping students’ engagement in language acquisition. In this sense, attitude is considered one of the key factors affecting second language acquisition, as it directly affects the level of effort learners devote to their language learning process (Gardner et al., 1985). Douglas (2000) reviewed numerous research studies on the effect of attitude on language learning and concluded that having a positive attitude toward oneself, the target language, and its community significantly contributes to language proficiency. Furthermore, learners who have a positive attitude toward language acquisition are more inclined to take on challenges and put forth effort (Karnchanachari, 2020; Tódor and Dégi, 2016). This aligns with previous research suggesting that attitude is a strong predictor of long-term success in language acquisition (Gardner et al., 1985; Dörnyei, 2005).

Effort is closely linked to motivation; increases in motivation are typically associated with higher effort (Richter et al., 2016). Motives play a central role in predicting effort (Mazeres et al., 2021). In the present study, intrinsic motivation emerged as a significant predictor of language learning effort, indicating that students who find learning English enjoyable and personally meaningful invest more time and sustained effort (Ryan and Deci, 2000). Intrinsic motivation has been consistently associated with successful language learning because it supports persistence and enthusiasm for learning activities (Douglas, 2000; Vallerand and Blssonnette, 1992). Such satisfaction-based engagement can strengthen commitment to learning over time (Ouahidi, 2020).

Similarly, the positive effect of extrinsic motivation on language learning effort indicates that students motivated by grades, recognition, professional opportunities, or social validation also show strong commitment to learning English. Although extrinsic motivation is sometimes considered less enduring than intrinsic motivation, it can still sustain engagement in highly structured academic settings (Noels et al., 2000). According to these results, improving students’ motivation and attitudes can have a significant effect on their learning effort and overall success when learning a foreign language.

Overall, both intrinsic and extrinsic motivation, together with positive attitudes toward foreign language learning, were associated with higher learning effort and success. By fostering positive attitudes and strong motivational frameworks, educators can help students sustain their commitment to language learning and enhance their proficiency development.

### 4.1 Limitations and future research

This study is subject to certain limitations that should be considered when interpreting the findings. The cross-sectional design limits causal inferences, and the reliance on self-reported data may introduce response bias. In addition, the sample, drawn from English-related departments, may not fully represent other student populations or cultural contexts. Future studies could employ longitudinal designs to examine changes over time, incorporate more diverse samples to enhance generalizability, and explore additional psychological or contextual variables—such as self-efficacy, classroom climate, or peer influence—to deepen the understanding of foreign language learning effort.

The findings of this study are expected to offer valuable insights for educational policymakers, curriculum developers, and curriculum implementers in understanding the broader dynamics of academic jealousy, motivation, and attitude toward language learning, and foreign language learning effort. Furthermore, this study is anticipated to serve as a guide for the implementation of targeted interventions and educational practices aimed at enhancing students’ overall engagement in the language learning process.

## Data Availability

The raw data supporting the conclusions of this article will be made available by the authors, without undue reservation.
